# From deep TLS validation to ensembles of atomic models built from elemental motions

**DOI:** 10.1107/S1399004715011426

**Published:** 2015-07-28

**Authors:** Alexandre Urzhumtsev, Pavel V. Afonine, Andrew H. Van Benschoten, James S. Fraser, Paul D. Adams

**Affiliations:** a Centre for Integrative Biology, Institut de Génétique et de Biologie Moléculaire et Cellulaire, CNRS–INSERM–UdS, 1 Rue Laurent Fries, BP 10142, 67404 Illkirch, France; b Faculté des Sciences et Technologies, Université de Lorraine, BP 239, 54506 Vandoeuvre-les-Nancy, France; c Physical Biosciences Division, Lawrence Berkeley National Laboratory, Berkeley, California, USA; d Department of Bioengineering and Therapeutic Sciences, University of California, San Francisco, San Francisco, CA 94158, USA; e Department of Bioengineering, University of California Berkeley, Berkeley, CA 94720, USA

**Keywords:** TLS model, TLS matrices, model validation, molecular mobility, ensemble of models, diffuse scattering, libration, vibration, correlated motion

## Abstract

The translation–libration–screw model first introduced by Cruickshank, Schomaker and Trueblood describes the concerted motions of atomic groups. Using TLS models can improve the agreement between calculated and experimental diffraction data. Because the **T**, **L** and **S** matrices describe a combination of atomic vibrations and librations, TLS models can also potentially shed light on molecular mechanisms involving correlated motions. However, this use of TLS models in mechanistic studies is hampered by the difficulties in translating the results of refinement into molecular movement or a structural ensemble. To convert the matrices into a constituent molecular movement, the matrix elements must satisfy several conditions. Refining the **T**, **L** and **S** matrix elements as independent parameters without taking these conditions into account may result in matrices that do not represent concerted molecular movements. Here, a mathematical framework and the computational tools to analyze TLS matrices, resulting in either explicit decomposition into descriptions of the underlying motions or a report of broken conditions, are described. The description of valid underlying motions can then be output as a structural ensemble. All methods are implemented as part of the *PHENIX* project.

## Abstract 2

Procedures are described for extracting the vibration and libration parameters corresponding to a given set of TLS matrices and their simultaneous validation. Knowledge of these parameters allows the generation of structural ensembles corresponding to these matrices.

## 1. Introduction   

### 1.1. Independent and concerted molecular motions   

It is currently difficult to derive a structural basis for concerted molecular motions from the models emerging from macromolecular crystallography, which describe each atom with a central position **r**_0_ and additional displacement parameters. Small-magnitude disorder (particularly thermal motion) can be captured by the Debye–Waller factor, which reflects the probability of an atom moving from its central position by a certain distance. If a model includes this approximation, the contribution of each atom to the structure factor (*h*, *k*, *l*) must be scaled by
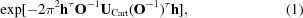
(see, for example, Grosse-Kunstleve & Adams, 2002 ▸ and references therein). Here, **O** is the orthogonalization matrix for the given crystal, **h** is the column vector of integer indices (*h*, *k*, *l*), **U**_Cart_ is an atomic displacement parameter (ADP) and the superscript τ stands for the matrix and vector transpose operation (here and in the following). [In Grosse-Kunstleve & Adams (2002 ▸) the orthogonalization matrix is defined as **A**; here, this letter is reserved for the matrix in the development of **U**_Cart_, following Tickle & Moss (1999 ▸).] The symmetric positive definite matrix **U**_Cart_ is defined by the average atomic shifts (and their correlations) along each coordinate axis. The matrix **U**_Cart_ varies between atoms and is diagonal (with equal elements) for atoms that are assumed to be moving isotropically.

**U**_Cart_ can accumulate contributions from several different sources, including overall crystal anisotropy (**U**_cryst_), various concerted motions (**U**_group_) and independent displacement of individual atoms (**U**_local_) (see, for example, Dunitz & White, 1973 ▸; Prince & Finger, 1973 ▸; Johnson, 1980 ▸; Sheriff & Hendrickson, 1987 ▸; Murshudov *et al.*, 1999 ▸; Winn *et al.*, 2001 ▸; , Dauter *et al.*, 2012 ▸; Afonine *et al.*, 2013 ▸).

Concerted motion contributing to **U**_group_ can be modelled by the translation–libration–screw approximation (TLS) introduced by Cruickshank (1956 ▸) and Schomaker & Trueblood (1968 ▸) and developed further in a number of publications, for example Johnson (1970 ▸), Scheringer (1973 ▸), Howlin *et al.* (1989 ▸, 1993 ▸), Kuriyan & Weis (1991 ▸), Schomaker & Trueblood (1998 ▸), Tickle & Moss (1999 ▸), Murshudov *et al.* (1999 ▸), Winn *et al.* (2001 ▸, 2003 ▸) and Painter & Merritt (2005 ▸, 2006*a* ▸,*b* ▸). This approximation is of special interest to structural biologists for two reasons. Firstly, TLS characterizes the anisotropic mobility of atomic groups and can provide insight into molecular mechanism. Secondly, it simplifies the crystallographic model by reducing the number of parameters while simultaneously providing a more realistic description of atomic displacements.

A common misconception of TLS parametrization is that its sole merit is to provide an economical method of accounting for anisotropic motions at low resolution. In fact, TLS parameterization can be useful regardless of the resolution of the available diffraction data. TLS has been successfully used to analyze functionally important molecular motions on several occasions (Kuriyan & Weis, 1991 ▸; Harris *et al.*, 1992 ▸; Šali *et al.*, 1992 ▸; Wilson & Brunger, 2000 ▸; Raaijmakers *et al.*, 2001 ▸; Yousef *et al.*, 2002 ▸; Papiz *et al.*, 2003 ▸; Chaudhry *et al.*, 2004 ▸), demonstrating that this approximation can provide critical structural information. However, the use of TLS models to derive functional insights is limited by the difficulty in analyzing the resulting motions. Although analysis of the resulting anisotropic displacement parameters is possible in some programs (Howlin *et al.*, 1993 ▸; Painter & Merritt, 2005 ▸), decomposing TLS models into structural ensembles comprised of many atomic models might enable more straightforward comparisons to other data sets, particularly in the case of diffuse X-ray scattering (Van Benschoten *et al.*, 2015 ▸). The major goal of this work is to develop an approach for translating TLS matrices into descriptions of corresponding molecular motions in terms of rotations and translations. In turn, this allows the validation of TLS parameters and the generation of structural ensembles. The latter will enable the broader use of TLS refinement for discovering and validating concerted molecular motions. In accomplishing this goal, we encountered several complications that suggest revisiting the fundamental processes of TLS refinement.

### 1.2. TLS model   

Since the displacement of a rigid group of atoms is a composition of translation and rotation (see, for example, Goldstein, 1950 ▸), Schomaker & Trueblood (1968 ▸) presented the matrices **U**_group,*n*_ for the concerted motion of a group of atoms *n* = 1, 2, … *N* as a sum, 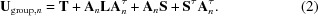
The antisymmetric matrices **A** *_n_* are functions of the Cartesian coordinates (*x_n_*, *y_n_*, *z_n_*) of atom *n*

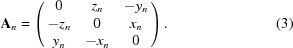
Matrix **S** and the symmetric matrices **T** and **L** are common to all atoms within each rigid group. **L** describes librations (oscillating rotations) around three mutually orthogonal rotation axes. **T** describes apparent translations of the atomic group (the term ‘vibrations’ might actually be more appropriate for random translations around a central position). **S** describes screw motions, *i.e.* the combination of librations and vibrations. We use the term ‘apparent translation’ because matrix **T** may have an additional contribution from librations as discussed in §[Sec sec2]2.

Thus, explicit information about atomic movement can be encoded into TLS matrices to produce inexplicit descriptors of motion. Both frameworks have merit: explicit description allows a straightforward interpretation and analysis of the motions, while the inexplicit TLS formalism provides a simpler framework for calculating structure factors. However, it is important to remember that TLS parameterization always arises from explicit atomic movement; thus, the TLS matrices should obey certain restrictions in order to be decomposed into structural ensembles representing concerted physical motions. Current refinement programs treat elements of the TLS matrices as independent variables with a constraint on the trace of the matrix **S** [tr(**S**); as discussed in §[Sec sec4]4] and post-refinement enforcement that the resulting **U**_group,*n*_ be non-negative definite (Winn *et al.*, 2001 ▸). As demonstrated below, enforcing **U**_group,*n*_ to be non-negative definite is not sufficient to guarantee that the refined TLS matrices are still consistent with an underlying physical model of concerted motion.

Previously, Zucker *et al.* (2010 ▸) analyzed all PDB entries containing TLS descriptions and suggested tools to validate the TLS parameters. However, this analysis focused exclusively on the ADP smoothness between neighbouring TLS groups. Failure to enforce all conditions on the individual components of **U**_group,*n*_, *i.e.* on the TLS matrices, may result in matrices that invalidate the TLS model. Using the methods and tools presented in this manuscript, we analyzed all structures from the PDB (Bernstein *et al.*, 1977 ▸; Berman *et al.*, 2000 ▸; about 105 000 entries, 25 000 of which contain TLS models, with a total of 200 000 sets of matrices). Our results demonstrate that significant issues are present in current TLS implementations. A third of the analyzed structures contain **T** or **L** matrices that are non-positive semidefinite and another third (Table 1 ▸) cannot describe libration–vibration correlated motions owing to the reasons discussed in §§[Sec sec2] [Sec sec3] [Sec sec4] [Sec sec5]2–5. Some of these errors (but not all) are trivial to fix, *e.g.* correcting marginally negative eigenvalues of **T** and **L** or modifying the trace of **S** (examples are given in §[Sec sec6]6 and in Table 1 ▸).

### 1.3. On the physical meaning and use of TLS   

Efforts to constrain TLS parameters to keep them physically meaningful have been discussed previously (Winn *et al.*, 2001 ▸; Painter & Merritt, 2006*a* ▸). It is universally accepted that *B* values need to be positive, occupancies must range between 0 and 1 and atomic coordinates should define model geometry in accordance with chemical knowledge. Similarly, provided that the TLS groups have been selected adequately, the TLS parameters describing the anisotropic harmonic motion of atomic groups (Schomaker & Trueblood, 1968 ▸) should be physically meaningful, otherwise TLS modelling may not be considered to be applicable. One such condition, but not the only one, is that the **T** and **L** matrices are positive semidefinite.

While calculating TLS matrices from corresponding libration and vibration parameters is rather straightforward (§[Sec sec2]2), the inverse procedure is less trivial. As discussed previously (Johnson, 1970 ▸; Scheringer, 1973 ▸; Tickle & Moss, 1999 ▸), the problem itself is poorly posed since the same set of diffraction data (and consequently the same set of TLS matrices) may correspond to different motions of the contributing atoms or atomic groups. Moreover, there are computational difficulties if all the conditions on the matrices have not been considered (§§[Sec sec3] [Sec sec4] [Sec sec5]3–5).

The set of TLS matrices corresponding to physically possible combinations of motions is obviously smaller than the set of all TLS matrices. Since restricting the parameter space of any function may inadvertently exclude a number of deep minima, including the global minimum, structural refinement that imposes conditions on TLS matrices may result in higher *R* factors than if these conditions were ignored. Since TLS modelling is an approximation to the true molecular motions that strongly depends on the assignment of TLS groups, lower *R* factors as result of using TLS may not always be indicative of this model being decomposable into a valid macromolecular motion.

### 1.4. Summary of the presented work   

In this article, we address the following points.

**Figure 1:**
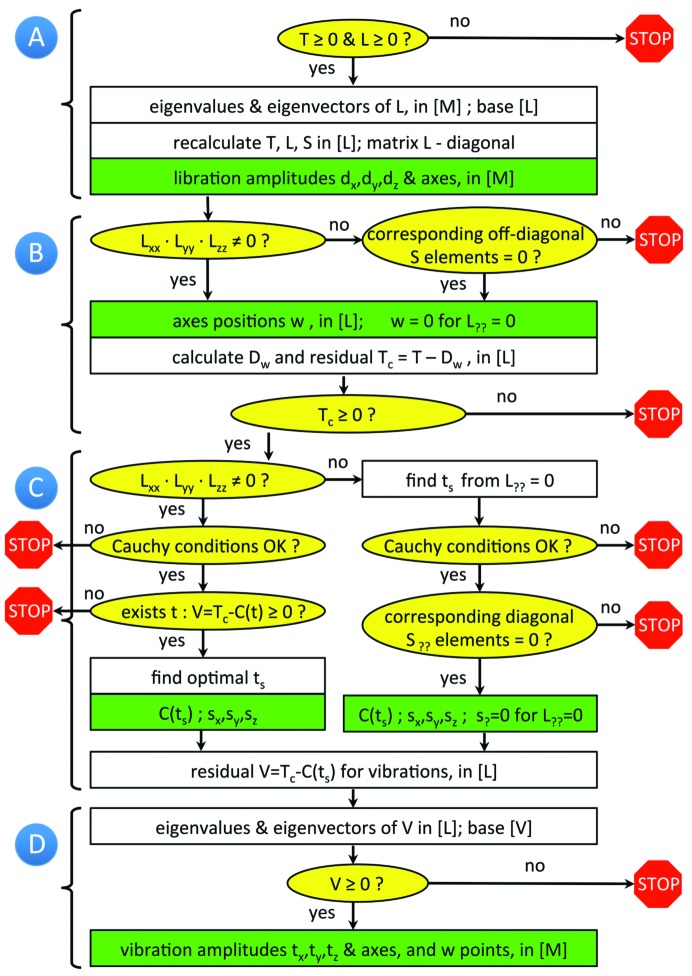
General flowchart of the TLS decomposition into libration and vibration composite motions. Yellow ellipses indicate conditions to be verified. Green rectangles indicate the output parameters of the composite motions. The letters *A*–*D* indicate different steps of the procedure as described in the text.

(i) We describe an algorithm (Fig. 1 ▸) that interprets the TLS matrices in terms of parameters of the corresponding motions. This includes the direction of the principal axes of vibration and libration, the corresponding root-mean-square displacements and the position of the libration axes, as well as the correlations between vibration and libration displacements.
(ii) We present a complete list of conditions that must be fulfilled to make the aforementioned TLS decomposition possible; this includes widely known conditions (*e.g.* **T** and **L** must be positive semidefinite) as well as a number of less trivial conditions that to the best of our knowledge have not been previously discussed.
(iii) We describe the calculation protocols in a ready-to-program style so that they can be implemented in existing or future software. Most of the calculations described in the manuscript are straightforward; less trivial expressions and proofs can be found in Appendix *A* [App appa] as well as in the review by Urzhumtsev *et al.* (2013 ▸).
(iv) We implemented the described algorithms in the open-source *Computational Crystallography Toolbox* (*cctbx*; Grosse-Kunstleve *et al.*, 2002 ▸). We also made two end-user applications available in the *PHENIX* suite (Adams *et al.*, 2010 ▸): *phenix.tls_analysis* for the analysis and validation of refined TLS matrices and their underlying motions and *phenix.tls_as_xyz* for generating ensembles of structures consistent with TLS matrices.
(v) We applied these programs to all PDB entries containing TLS matrices. We discovered that the majority of these matrices cannot describe motions. In a number of cases a marginal modification of the TLS matrices can correct the errors.
(vi) We used *phenix.tls_as_xyz* to generate a predicted structural ensemble for the calculation of X-ray diffuse scattering from the glycerophosphodiesterase GpdQ (Van Benschoten *et al.*, 2015 ▸).

## 2. Calculating TLS matrices from elemental motions   

This section provides a step-by-step protocol for calculating TLS matrices from the parameters of the composite vibrations and librations. Inverting this scheme provides a method of extracting libration/vibration parameters from the TLS matrices.

### 2.1. Constructing TLS matrices from the parameters of the libration and vibration   

The matrices in (2) depend on the basis in which the atomic coordinates are given. We use an index in square brackets to indicate which basis is used. Let the atoms be given in some basis denoted [M]; for example, it may be the basis corresponding to the model deposited in the PDB. Even if a rigid group is involved in several simultaneous motions (assuming that the amplitudes of these motions are relatively small and the motions are harmonic), the total motion can be described by a libration around three axes **l**_*x*_, **l**_*y*_, **l**_*z*_ that are mutually orthogonal and by a vibration along three other mutually orthogonal axes, **v**_*x*_, **v**_*y*_, **v**_*z*_. These triplets of axes form the other two bases, [L] and [V].

In (2) the matrix **T** is a sum of several components. In the absence of librations (that is, matrices **L** and **S** are zero) it is equal to the contribution **V** arising from pure vibrations. In the basis [V] this matrix is diagonal,
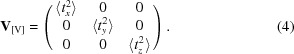
Here, 〈*t_x_* ^2^〉, 〈*t_y_* ^2^〉, 〈*t_z_* ^2^〉 are the corresponding squared root-mean-square deviations (r.m.s.d.s) along the principal vibration axes **v**_*x*_, **v**_*y*_, **v**_*z*_ and are expressed in Å^2^. If there are librations, the matrix **L** is always diagonal in the basis [L], 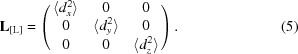
Here, 〈*d_x_* ^2^〉, 〈*d_y_* ^2^〉, 〈*d_z_* ^2^〉 are the squared r.m.s.d.s of the vibration angles expressed in squared radians; for small deviations they are numerically equal to the squared r.m.s.d.s of points at a unit distance from the corresponding axes.

In reality, the principal vibration and libration axes are not parallel to each other; practically, it is convenient to express the matrices in a common basis. Basis [L] is more convenient for this since in this basis the elements of **S** (see below) are easily expressed through geometric parameters of librations. Matrix **V** in this basis is no longer diagonal but is instead equal to
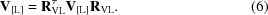
Here, **R**_VL_ is the transition matrix that describes the rotation superposing the vectors **v**_*x*_, **v**_*y*_, **v**_*z*_ with the vectors **l**_*x*_, **l**_*y*_, **l**_*z*_ (Appendix *A* [App appa]). Frequently, vibration and libration motions are not independent but instead are correlated to form screw rotations. It is convenient to characterize screw rotations by the parameters *s_x_*, *s_y_*, *s_z_*: for a screw rotation by *d_z_* radians around an axis parallel to **l**_*z*_ each atom is shifted by *s_z_* Å along this axis. A similar definition is used for the other two parameters. If the axes pass through the origin, such a correlation generates an additional contribution **C**_[L]_ to the **T** matrix that arises from screw motions, 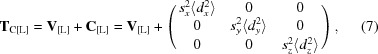
and also results in a nonzero **S** matrix,
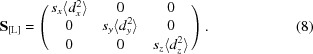
Finally, the principal libration axes do not necessarily pass through the origin, or even have a common point (*i.e.* they may not intersect). If they pass through the points **w** ^*lx*^_[L]_ = (*w_x_^lx^*, *w_y_^lx^*, *w_z_^lx^*), **w** ^*ly*^_[L]_ = (*w_x_^ly^*, *w_y_^ly^*, *w_z_^ly^*), **w** ^*lz*^_[L]_ = (*w_x_^lz^*, *w_y_^lz^*, *w_z_^lz^*), respectively, this generates an additional component to the **T** matrix, 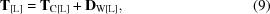
where
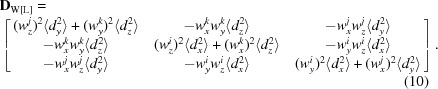
Taking into account both the screw motion and the position of the libration axes, the matrix **S** becomes
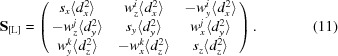
Finally, the matrices in the original basis [M] where they are reported together with the atomic coordinates are obtained from **L**_[L]_ (5), **T**_[L]_ (9), **S**_[L]_ (11) as
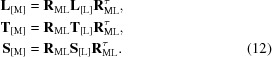
Here, **R**_ML_ is the transition matrix from the basis [M] to the basis [L] (Appendix *A* [App appa]).

### 2.2. Molecular basis and centre of reaction   

The TLS matrices also depend on the choice of the origin. Clearly, the coordinates of the position of the libration axes change as function of the origin. Usually, the origin is taken to be the centre of mass of the atomic group or the point where the mean atomic displacements are similar in magnitude to each other owing to librations around each of the principal axes. This second point is called the centre of diffusion (Brenner, 1967 ▸) or the centre of reaction (Tickle & Moss, 1999 ▸). Choosing the origin at the centre of reaction minimizes the trace of **T** and makes **S** symmetric (Brenner, 1967 ▸; Tickle & Moss, 1999 ▸; Urzhumtsev *et al.*, 2013 ▸). Shifting from one origin to another changes **T** and **S** but does not change **L** and does not modify the algorithm of the search for the composite motions. In the following, we consider the matrices to be in their original basis (for example, as they are defined in the PDB).

## 3. Calculating elemental motions from TLS matrices: libration axes   

This section provides a step-by-step explanation of the inverse problem, *i.e.* calculating the vibration and libration axes and the corresponding r.m.s.d.s, the position of the libration axes and the parameters describing the correlations between librations and vibrations from given TLS matrices.

### 3.1. Diagonalization of the **L** matrix ([L] basis; step *A*)   

Suppose that we know the elements of the matrices (12) in the basis [M]. By construction, the matrices **T** and **L** should be positive semidefinite (Appendix *B* [App appb]) and symmetric, *T*_[M]*xy*_ = *T*_[M]*yx*_, *T*_[M]*xz*_ = *T*_[M]*zx*_, *T*_[M]*yz*_ = *T*_[M]*zy*_ and *L*_[M]*xy*_ = *L*_[M]*yx*_, *L*_[M]*xz*_ = *L*_[M]*zx*_, *L*_[M]*yz*_ = *L*_[M]*zy*_. These properties hold for any rotation of the coordinate system, *i.e.* in any Cartesian basis; this is important for further analysis of the **T** matrices.

We start the procedure from the matrix **L**_[M]_, which depends only on the libration parameters. The principal libration axes correspond to its three mutually orthogonal eigenvectors. Firstly, we search for the corresponding eigenvalues 0 ≤ λ_1_ ≤ λ_2_ ≤ λ_3_, which must be non-negative (see equation 5; eigenvalues do not change with the coordinate system). Let **l**_1_, **l**_2_, **l**_3_ be the corresponding normalized eigenvectors from which we construct a new basis [L] as
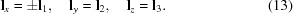
The appropriate sign for **l**_*x*_ is chosen so that the vectors in (13) form a right-hand triad; for example, one can take **l**_*x*_ = **l**_*y*_ × **l**_*z*_ which guarantees such a condition. The TLS matrices in the [L] basis are
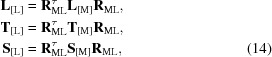
where **R**_ML_ is the transition matrix from basis [M] into basis [L] (Appendix *A* [App appa]). In this new basis, matrix **L**_[L]_ is diagonal with elements *L*_[L]*xx*_ = λ_1_, *L*_[L]*yy*_ = λ_2_, *L*_[L]*zz*_ = λ_3_, giving the estimates 〈*d_x_* ^2^〉 = *L*_[L]*xx*_, 〈*d_y_* ^2^〉 = *L*_[L]*yy*_, 〈*d_z_* ^2^〉 = *L*_[L]*zz*_ of the squared libration amplitudes around the three principal libration axes.

### 3.2. Position of the libration axes in the [L] basis (step *B*)   

In the basis [L] the libration axes are parallel to the co­ordinate axes but do not necessarily coincide with them. Let them pass through some points **w** ^*lx*^, **w** ^*ly*^, **w** ^*lz*^, respectively, that must be identified. Using (11), we calculate the coordinates of these points as
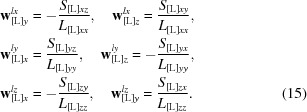
A zero value for any denominator in (15) means that there is no rotation around the corresponding axis; in this case, the two corresponding numerator values must also be equal to zero and thus assign zero values to the corresponding coordinates in (15). Otherwise, the input matrices are incompatible and the procedure must stop (Appendix *B* [App appa]). The *x* component of **w** *^lx^*, the *y* component of **w** *^ly^* and the *z* component of **w** *^lz^* in the basis [L] can be any values. For presentation purposes, it might be useful to assign them as
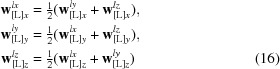
that will position each of these points in the middle of the two other axes. This choice also reduces eventual rounding errors.

Knowing the positions (15 and 16) of the libration axes and elements of **L**_[L]_, we can calculate the contribution **D**_W[L]_ (10) from an apparent translation owing to the displacement of the libration axes from the origin. Then, by inverting (9) we can calculate the residual matrix **T**_C[L]_ after removal of this contribution, 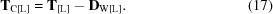
Matrix (17) must be positive semidefinite (Appendix *B* [App appb]) as it is a sum (7) of two positive semidefinite matrices. Matrices **S**_[L]_ and **L**_[L]_ are not modified at this step.

## 4. Calculating elemental motions from TLS matrices: screw components (step *C*)   

### 4.1. Correlation between libration and vibration and a choice of the diagonal elements of **S**   

Next, we use the matrices **L**_[L]_ and **S**_[L]_ to determine the screw parameters *s_x_*, *s_y_*, *s_z_*, remove the screw contribution from the **T**_C[L]_ matrix using (7) and (17) and finally extract the matrix **V**_[L]_ for uncorrelated vibrations. However, there is an ambiguity in the definition of **S**_[L]_ which is apparent from the observation that the matrices **U**_concerted,*n*_ of individual atoms will not change if the same number *t* is added or removed simultaneously from all three diagonal elements of **S**_[L]_. This is usually known as indetermination of the trace of this matrix (Schomaker & Trueblood, 1968 ▸). A current practice (an illustration is provided in §[Sec sec6.1]6.1) is to choose *t* such that it minimizes the trace (rather its absolute value) of the resulting matrix,
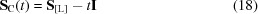
(where *I* is a unit matrix), *i.e.* minimizing vibration–libration correlation (Urzhumtsev *et al.*, 2013 ▸), or simply makes the trace equal to zero (http://www.ccp4.ac.uk/html/restrain.html; Coppens, 2006 ▸). The unconditioned minimization
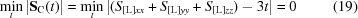
gives
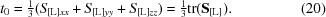
However, this value may lead to matrices for which libration–vibration decomposition is impossible and, in particular, prohibits the generation of structural ensembles. For example, if the elements of matrix **S** and the corresponding values *s_x_*, *s_y_*, *s_z_* are too large, the matrix **V** in (7) may be not positive definite for a given **T**_C[L]_. The next sections describe a procedure that defines the constraints on the diagonal elements of matrix **S** when using (18).

### 4.2. Cauchy–Schwarz conditions   

After removing **D**_W[L]_ (17), the set of matrices **T**_C[L]_, **L**_[L]_ and the matrix **S**_[L]_ with the removed off-diagonal elements (reducing the matrix in equation 11 to the form in equation 8) correspond to a combination of vibrations with screw rotations around the axes crossing the origin. The diagonal elements of these matrices must satisfy the Cauchy–Schwarz inequality (Appendix *A* [App appa]), 
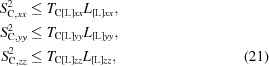
that in turn defines the conditions (Appendices *A* [App appa] and *B* [App appb])
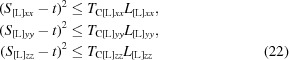
or
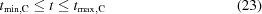
with
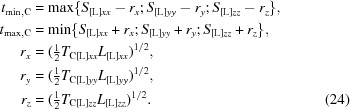
In particular, this unambiguously defines the *t* value if one of the diagonal elements of the matrix **L**_[L]_ is zero so that the trace of **S**_[L]_ cannot be changed or assigned arbitrarily (see §[Sec sec4.4]4.4).

### 4.3. Positive semidefinition of the **V** matrix   

The last condition to check is that the matrix **V** is positive semidefinite. Let us suppose that all diagonal elements of the matrix **L**_[L]_ are different from zero; §[Sec sec4.4]4.4 considers the alternative case. From (5), (7), (8) and (18) we find the expression for the screw contribution
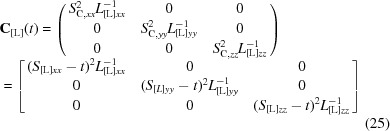
to be subtracted from matrix (17) as
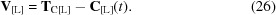
Matrix **V**_[L]_ is positive semidefinite along with
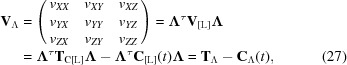
where
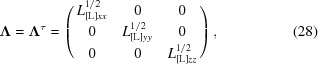


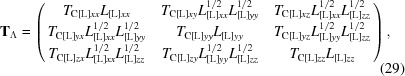


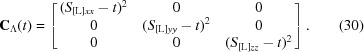
Necessarily, all diagonal terms of (30) cannot be larger than the maximal eigenvalue τ_max_ of matrix (29), giving a necessary condition (Appendix *B* [App appb])
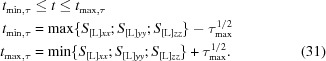
The condition that all diagonal terms of (30) are not larger than the minimum eigenvalue τ_min_ of (29) is sufficient but not necessary.

Matrix **V**_Λ_ is positive semidefinite if and only if all three of its real eigenvalues are non-negative (some of them may coincide with each other). They are the roots of the cubic characteristic equation
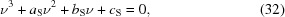
with the coefficients
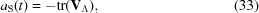


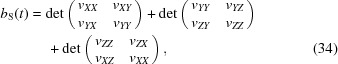


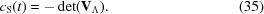
The roots of (32) are positive if and only if the three inequalities below hold simultaneously,
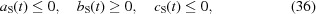
where the left parts are polynomials of order two, four and six of the parameter *t*, all with the unit highest-order coefficient (Appendix *A* [App appa]). The first condition in (36) defines the interval for *t* values (Appendix *B* [App appb]),
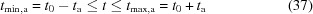
with
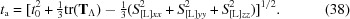
We failed to find analytical expressions corresponding to the two other inequalities. As a result, the following numerical procedure is suggested to find the best *t* value that is physically acceptable.

(i) Calculate the *t*_0_ value (20).
(ii) Calculate the interval (*t*_min_, *t*_max_) for allowed *t* values as the intersection of intervals (23), (31) and (37), *t*_min_ = max{*t*_min,C_, *t*_min,τ_, *t*_min,a_}, *t*_max_ = min{*t*_max,C_, *t*_max,τ_, *t*_max,a_}; if *t*_min_ > *t*_max_ the problem has no solution and the procedure stops (Appendix *B* [App appb]).
(iii) If *t*_min_ = *t*_max_ we check the conditions *b*_S_(*t*_min_) ≥ 0, *c*_S_(*t*_min_) ≤ 0, or the condition that **V**_Λ_ is positive semidefinite; if the conditions are satisfied we assign *t*_S_ = *t*_min_, otherwise the problem has no solution and the procedure stops (Appendix *B* [App appb]).
(iv) If *t*_min_ < *t*_max_ we search numerically, in a fine grid, for the point *t*_S_ in the interval (*t*_min_, *t*_max_) and closest to *t*_0_ such that *b*_S_(*t*_S_) ≥ 0, *c*_S_(*t*_S_) ≤ 0; if for any point of this interval at least one of these inequalities is wrong then the procedure stops (Appendix *B* [App appb]).
(v) We accept the value obtained at the step (iii) or (iv) as the final *t*_S_.

### 4.4. Singular sets of rotation   

When one of the *L*_[L]*xx*_, *L*_[L]*yy*_, *L*_[L]*zz*_ values is zero (that is, there is no rotation around the corresponding axis), straightforward use of the standard procedure including (25) becomes impossible. However, in this case the *t*_S_ value must be equal to *S*_[L]*xx*_, *S*_[L]*yy*_ or *S*_[L]*zz*_, corresponding to the axes with no rotation, making the corresponding diagonal element in (25) equal to zero and turning the corresponding inequality in (24) into an equality. For example, if *L*_[L]*xx*_ = 0 then *t*_S_ = *S*_[L],*xx*_, resulting in *C*_[L]*xx*_ = 0. We simply need to check two other conditions in (21) and confirm that the residual matrix is positive semidefinite (for example, by calculating equation 36). If *t*_S_ does not satisfy these conditions, the problem has no solution (Appendix *B* [App appb]).

### 4.5. Screw parameters   

For the *t* = *t*_S_ determined above we calculate the matrix **S**_C_(*t*_S_) (18). From this matrix we obtain the screw parameters *s*_*x*_ = *S*_C,*xx*_ *L* ^−1^_[L]*xx*_, *s*_*y*_ = *S*_C,*yy*_ *L* ^−1^_[L]*yy*_, *s*_*z*_ = *S*_C,*zz*_ *L* ^−1^_[L]*zz*_ for the rotation axes currently aligned with the coordinate axes of the basis [L]. If one of the *L*_[L]*xx*_, *L*_[L]*yy*_, *L*_[L]*zz*_ values is equal to zero, the corresponding diagonal element of **S**_C_ must also be equal to zero, and we assign the corresponding screw parameter, *s*_*x*_, *s*_*y*_ or *s*_*z*_, to be zero. Otherwise, the matrices are inconsistent with each other and the procedure stops (Appendix *B* [App appb]).

## 5. Calculating elemental motions from TLS matrices: vibration components (step *D*)   

### 5.1. Matrix **V** and vibration parameters in [L] basis   

For the known *t*_S_, the matrices **C**_[L]_(*t*_S_) and then **V**_[L]_ are calculated using (25) and (26). The parameter values of the independent vibrations are calculated from the **V**_[L]_ matrix similarly to those for the independent librations, as we obtain them from **L**_[M]_. Firstly, we calculate the three eigenvalues 0 ≤ μ_1_ ≤ μ_2_ ≤ μ_3_ of matrix **V**_[L]_ (Appendix *B* [App appb]; in practice, all of them are strictly positive). We then identify three corresponding unit eigenvectors **v**_1_, **v**_2_, **v**_3_ that are orthogonal to each other and assign
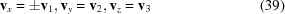
[the sign for **v**_*x*_ is taken so that the vectors (39) form a right-hand triad]. We remind the reader that these axes define the basis [V] in which matrix **V**_[V]_ (6) is diagonal with elements *V*_[V]*xx*_ = μ_1_, *V*_[V]*yy*_ = μ_2_, *V*_[V]*zz*_ = μ_3_. This defines the last missing parameters, namely the values of the squared r.m.s.d.s along these axes: 〈*t_x_* ^2^〉 = *V*_[V]*xx*_, 〈*t_y_* ^2^〉 = *V*_[V]*yy*_, 〈*t_z_* ^2^〉 = *V*_[V]*zz*_.

### 5.2. Vibration and libration axes in [M] basis   

The libration and vibration amplitudes and screw parameters are independent of the choice of the basis, and the direction of the libration axes is known in the principal [M] basis. However, the directions of the uncorrelated translations **v**_1_, **v**_2_, **v**_3_ that were calculated in §[Sec sec4]4 and the points **w** ^*lx*^_[L]_, **w** ^*ly*^_[L]_, **w** ^*lz*^_[L]_ belonging to the libration axes (§[Sec sec3.2]3.2) are now known in the [L] basis.

To obtain the coordinates (*w* ^*lx*^_[M]*x*_, *w* ^*lx*^_[M]*y*_, *w* ^*lx*^_[M]*z*_), (*w* ^*ly*^_[M]*x*_, *w* ^*ly*^_[M]*y*_, *w* ^*ly*^_[M]*z*_), (*w* ^*lz*^_[M]*x*_, *w* ^*lz*^_[M]*y*_, *w* ^*lz*^_[M]*z*_) of these points in the [M] basis, we apply the transformation
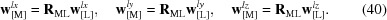
Similarly, the vectors defining the direction of the axes **v**_*x*_, **v**_*y*_, **v**_*z*_ in the basis [M] can be obtained as
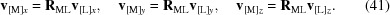
This step finalizes the extraction of the parameters of the motions that correspond to the given set of TLS matrices. §[Sec sec6]6 provides some examples of this procedure applied to models deposited in the PDB. §[Sec sec7]7 describes an example in which knowledge of the motion parameters extracted from the TLS matrices is necessary to explicitly simulate the ensemble of corresponding structures and the corresponding X-ray diffuse scattering.

## 6. Examples of TLS matrix analysis   

As discussed in §[Sec sec1]1, there are numerous examples of fruitful application of the TLS formalism to structural studies of concerted motion. The goal of this section is to illustrate the algorithm described above, to describe possible traps that emerge during refinement and to discuss further developments.

### 6.1. Survey of available TLS matrices in the PDB   

**Figure 2:**
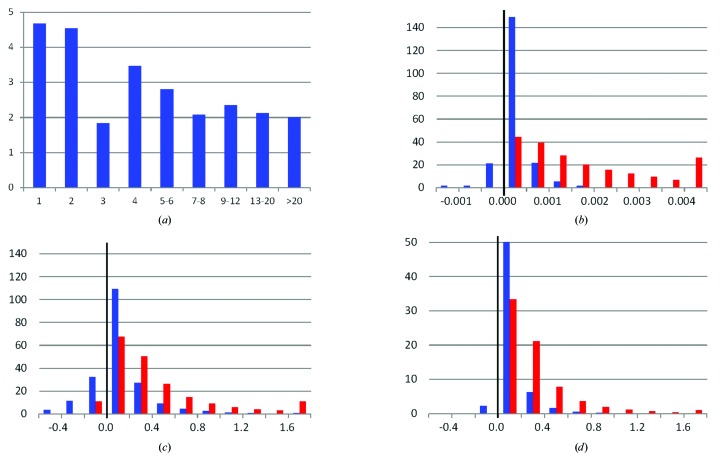
The number of PDB entries (in thousands) as a function of various parameters. The blue histogram in (*b*), (*c*) and (*d*) is for the minimum eigenvalue and the red histogram is for the maximum eigenvalue. The leftmost and rightmost bins include all cases with values less than or greater than the limits given on the axis, respectively. The eigenvalues are given in rad^2^ for **L** and in Å^2^ for **T**. The total number of TLS groups is 203 261 for (*a*), (*b*) and (*c*) and about 70 000 for (*d*) when the matrix **V** could be calculated. (*a*) The number of TLS groups per entry; the largest is 283. (*b*) Distribution of eigenvalues of the matrix **L**; the minimum eigenvalue varies from −0.285 to 0.164 and the maximum eigenvalue varies from −0.001 to 0.409. (*c*) Distribution of eigenvalues of the matrix **T**; the minimum eigenvalue varies from −20.716 to 6.852 and the maximum eigenvalue varies from −1.551 to 28.676. (*d*) Distribution of eigenvalues of the matrix **V** (the **S** matrix optimized as described in the article); the minimum eigenvalue varies from −0.001 to 2.815 and the maximum eigenvalue varies from 0 to 5.950.

We have analyzed all available TLS matrices in the PDB. From an overall 106 761 entries (as of March 2015), 25 904 use TLS modelling. More than 20 000 of these entries have several TLS groups, resulting in a total of 203 261 sets of TLS matrices (Fig. 2 ▸ *a*), with the largest number of groups per entry being 283 (PDB entry 3u8m; Rohde *et al.*, 2012 ▸). About a third of these sets have negative eigenvalues for the deposited **T** or **L** matrices. Some of these values are only slightly negative (Figs. 2 ▸ *b* and 2 ▸ *c*) and can be considered to be rounding errors, while the worst values are as small as −0.28 rad^2^ for **L** and −20.72 Å^2^ for **T**. For 11 412 **T** matrices and 138 **L** matrices all three eigenvalues are negative.

Another third of the TLS groups cannot be interpreted by elemental motions owing to other reasons, as described in §§[Sec sec3]3 and [Sec sec4]4 (Table 1 ▸).

After an initial screen to find the positive definite **T** and **L** matrices, we then ran a search for the elemental motions in two modes. Firstly, we tried to decompose the TLS matrices as taken directly from the PDB files. As expected, the average value of tr(**S**) is 3 × 10^−5^ Å (*i.e.* practically zero) and the corresponding r.m.s.d. is σ = 10^−2^ Å. About 120 000 **S** matrices have |tr(**S**)| < 10^−4^ Å. The numbers of matrices with |tr(**S**)| larger than 1σ, 3σ, 10σ and 20σ are only 3772, 486, 31 and three, respectively. We then applied the aforementioned algorithm with the optimal choice of the value *t*_S_ to be subtracted from the diagonal **S** elements in each case.

Table 1 ▸ shows the results of both runs and illustrates that it is possible to fix the problems found in 6500 of the TLS sets (corresponding to about 500 PDB entries) by a correction of the diagonal elements of the **S** matrix as described above. The table takes into account possible rounding errors by correcting slightly negative eigenvalues (those closer in value to zero than 10^−5^ of the default units: Å^2^, rad^2^ and Å rad for **T**, **L** and **S**, respectively). For example, when running the algorithm in the **S** optimizing mode the program can formally calculate the **V** matrix for about 70 000 sets. For 2296 cases this matrix has negative eigenvalues (Fig. 2 ▸ *d*), while in 2294 cases these eigenvalues are closer to 0 than 10^−5^ Å^2^; for such matrices the program makes automatic corrections and continues the process.

It is important to note that even if the parameters of the elemental motions can be formally extracted from the TLS matrices, this does not guarantee that they will make physical sense and therefore be valid for decomposition into a representative structural ensemble. Clearly, vibration amplitudes on the order of 20 Å^2^ cannot represent harmonic vibrations (Fig. 2 ▸ *d*). Similarly, the linear rotation approximation contained in TLS theory is valid only up to approximately 0.1 rad; however, much larger values can be found in the PDB (Fig. 2 ▸ *b*). Similar restrictions also hold for the screw parameters. The products *s_x_d_x_*, *s_y_d_y_*, *s_z_d_z_* show the mean shifts along the screw axes owing to librations around these axes; the values found in the PDB approaching 3 Å seem to be too large to describe harmonic motions.

For a more detailed analysis, we selected several entries from the PDB. For each structure, we applied a standard TLS refinement protocol as implemented in *phenix.refine* (Afonine *et al.*, 2012 ▸) including automatic determination of the TLS groups. During refinement, 20 matrix elements were refined independently, six for **T**, six for **L** and eight for **S**; the three diagonal elements of **S** were constrained such that the trace of the matrix is equal to 0. The procedure described above (§§[Sec sec3] [Sec sec4] [Sec sec5]3–5) was then applied to all sets of obtained TLS matrices.

We remind the reader that the elements of the **L** and **S** matrices are expressed in rad^2^ and Å rad, while in the PDB files they are in deg^2^ and in Å deg, respectively (Table 2 ▸).

### 6.2. Synaptotagmin   

**Figure 3:**
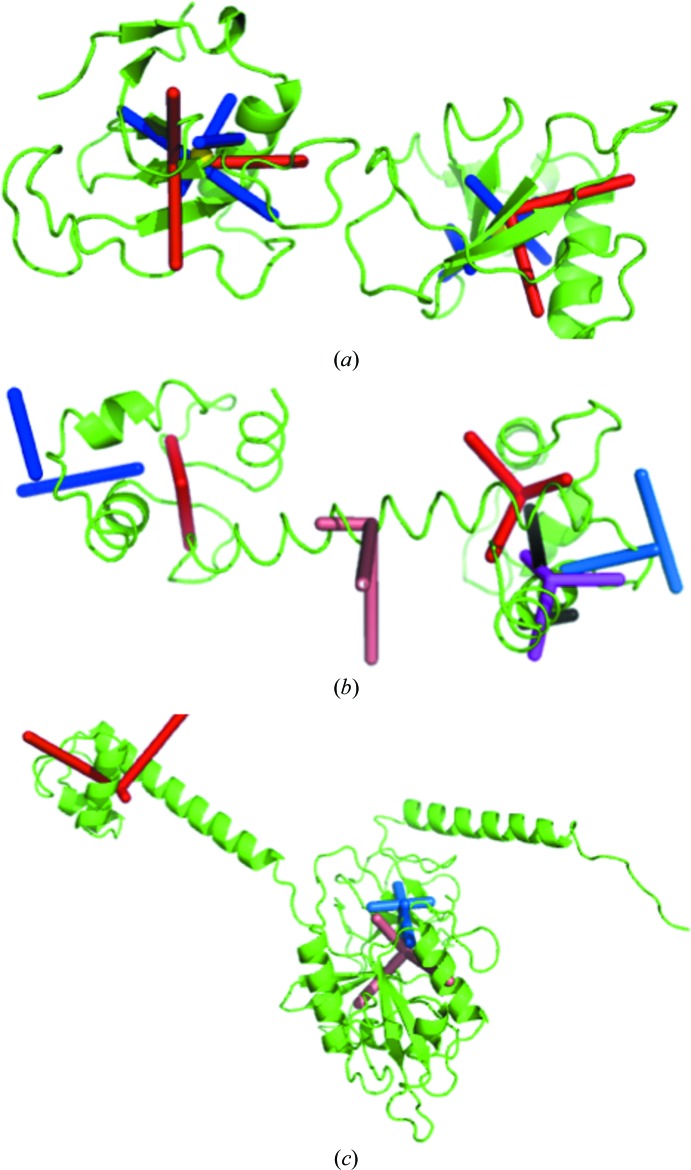
Examples of the vibration–libration ensembles. Red/salmon/magenta sticks indicate the principal vibration axes, with the origin in the centre of the group; blue/marine/black sticks are the libration axes. Yellow spheres in the 1dqv model show the reaction centres. (*a*) 1dqv model. (*b*) 1exr model; note pure vibrations for group 3 (the helix) and the absence of one of the libration axes for groups 1 and 2. (*c*) 4b3x model. Libration axes for the first group are not shown as they are too far from the molecule.

The crystals of synaptotagmin III (PDB entry 1dqv; Sutton *et al.*, 1999 ▸) contain two copies of the molecule in the asymmetric unit. The structure after re-refinement by *phenix.refine* without TLS modelling has an *R*_work_ of 0.200 and an *R*_free_ of 0.231 at a resolution of 2.5 Å. Performing TLS refinement with each molecule taken as a single TLS group reduced the *R* factors to *R*_work_ = 0.177 and *R*_free_ = 0.211, indicating that this additional modelling significantly improves the agreement with the experimental data. Table 2 ▸ shows the two sets of matrices and Table 3 ▸ contains the corresponding motion parameters extracted using our approach. For the two groups both vibrations and librations are practically isotropic and are of the same order of magnitude. Fig. 3 ▸(*a*) shows the principal axes of these motions.

### 6.3. Calmodulin   

The structure of calmodulin (PDB entry 1exr; Wilson & Brunger, 2000 ▸) has been determined previously at a resolution of 1.0 Å. This example illustrates possible problems that can be solved by a minimal correction of the TLS values. For re-refinement with *phenix.refine* the model was automatically split into four TLS groups. For the first group, one of the eigenvalues of the matrix **L** was equal to −2 × 10^−5^ rad^2^. If we consider this value to be zero (in this case the zero value must be also assigned to off-diagonal elements of the first row of the matrix **S**), the composite motions contain only two libration axes and their parameters can be extracted. Corresponding modifications of the resulting matrices **U**_group,*n*_ (2) can be compensated for by respective adjustment of the individual contributions **U**_local,*n*_. This keeps the total ADP parameters **U**_Cart,*n*_ unchanged, thus maintaining the previously calculated structure factors and *R* factors. An accurate separation of total atomic displacement parameter values into contributions from several sources (see, for example, Murshudov *et al.*, 1999 ▸; Winn *et al.*, 2001 ▸, 2003 ▸; Afonine *et al.*, 2012 ▸) is a separate ongoing project (Afonine & Urzhumtsev, 2007 ▸).

For the second TLS group, the refined TLS matrix elements contained one degenerate libration. The procedure described in §§[Sec sec3] [Sec sec3] [Sec sec3]3–5 was successfully applied. Note that this procedure modified the diagonal elements of the matrix **S**, removing an appropriate value of the parameter *t*_S_ (§[Sec sec4.4]4.4) and making tr(**S**) nonzero.

For the third group, all three eigenvalues of the matrix **L** were extremely small (0.0, 0.8 × 10^−5^ and 3 × 10^−5^ rad^2^), producing high computational instability and extremely large screw parameters that resulted in the inability of the procedure to find a positive semidefinite **V**_[L]_ (27). If we define all librations to be absent and replace matrix **L** (and respectively **S**) by zero matrices, the vibration parameters can easily be found from **T**. In fact, this TLS group is a helix held at both ends by large domains, which leads to the expectation of a pure vibration motion.

Finally, for the fourth group one of the diagonal elements of the matrix **T** was marginally negative. Increasing all of the diagonal elements of the matrix **T** by 0.002 Å^2^ makes this matrix positive definite (this corresponds to *B* = 0.16 Å^2^). As discussed above, this adjustment can be compensated for by removing the equivalent amount from individual atomic contributions **U**_local,*n*_ (such a subtraction keeps the individual atomic contributions positive). This group vibrates in a plane (Fig. 3 ▸ *b*) and the principal vibration axis of group 3 (the helix) is parallel to this plane, leading to the plausible hypothesis that groups 3 and 4 at least partially move together or slide along each other.

To check the influence of the manual modification on the TLS matrices, we recalculated the *R* factors before and after performing these changes without updating the individual atomic contributions **U**_local,*n*_. For all of the modifications described above, including the ensemble of modifications applied together, the *R* factors only varied in the fourth significant digit.

This example demonstrates that although current refinement procedures may result in TLS matrices that are unable to satisfy the previously mentioned conditions, small changes to them may provide sufficient correction. This highlights the need to use appropriate restraints or constraints on refinable parameters within the TLS model.

### 6.4. Initiation translation factor 2 (IF2)   

The structure of IF2 (PDB entry 4b3x) has recently been solved in one of our laboratories (Simonetti *et al.*, 2013 ▸) with an *R*_work_ of 0.180 and an *R*_free_ of 0.219 at a resolution of 1.95 Å. *A posteriori* TLS refinement was performed with two groups: the first group included the N-terminus and the following long helix, and the second included the rest of structure. Re-refining the model produced better statistics, with *R*_work_ = 0.176 and *R*_free_ = 0.203. In this example, the TLS matrices from the first group were not directly interpretable because the residual matrix **V**_[L]_ was not positive semidefinite (the minimal eigenvalue was −0.05). Similarly to the last TLS group in calmodulin, we artificially added 0.06 Å^2^ to all diagonal elements of the matrix **T**, corresponding to roughly 5 Å^2^ (the same amount has been removed from the residual atomic *B* values, thus leaving the *R* factors unchanged). This correction allowed interpretation of the TLS matrices in terms of elemental motions. We note that for the first TLS group one of the rotations was degenerate and that the assignment tr(**S**) = 0 would make this matrix incompatible with **L**. Table 3 ▸ shows that the vibrations of this group are essentially anisotropic. Fig. 3 ▸(*c*) also shows that the libration axes for this group pass quite far away from the molecule, which makes the corresponding rotation similar to a translation. Additionally, we believe that the large *s*_*z*_ value indicates that the matrix **S** is not well defined. The matrices for the second group were interpreted and revealed isotropic vibrations and librations.

Finally, we tried to apply the same procedure after choosing the TLS groups manually as residues 1–50 (N-terminus), 51–69 (helix), 70–333 (G domain) and 343–363 (the connector to the C domain, which is absent in this structure). As before, the matrices were interpretable for the G domain. For groups 2 and 4, after an adjustment similar to those discussed above (a slight increase of the diagonal **T** elements with a decrease of the residual atomic *B* values), we obtained a pure vibration for the helix (as for the calmodulin case) and a libration around a single axis for the terminal group. In contrast, we failed to find reasonably small corrections for the matrices of the first group that would make them interpretable in terms of physical motions that in particular can be represented by a structural ensemble.

This case exemplifies a situation in which the current TLS refinement protocols result in matrices that significantly reduce the *R* factors without providing refined TLS parameters that can be decomposed into a physically realistic motion of one of the groups. This highlights the need to improve TLS refinement algorithms by making use of constraints on aforementioned conditions on TLS matrices.

## 7. Interpreting TLS matrices with a structural ensemble   

### 7.1. Generation of an explicit set of atomic models with a variability consistent with TLS   

Some structural problems may explicitly require a set of models that describe a given mobility, for example corresponding to the TLS matrices for harmonic motion. An example of such a problem is described in the accompanying paper by Van Benschoten *et al.* (2015) (and is briefly presented in §[Sec sec7.4]7.4), in which X-ray diffuse scattering data were compared with calculated data corresponding to different types of molecular motion. Other examples may include analyzing larger-scale anharmonic motions, for which techniques such as molecular-dynamics trajectories have traditionally been used (McCammon *et al.*, 1977 ▸).

If a model deposited in the PDB contains TLS matrices, the matrices can be decomposed as described above. As soon as a combination of vibrations and librations is extracted from the TLS matrices, we can explicitly build a corresponding set of models. Knowledge of the three vibrations and three librations provides the atomic shifts underlying the total displacement.

It is generally more convenient to generate each group of atomic shifts in its own basis: shifts Δ^*V*^_[V]_ **r**_*n*_ owing to vibration in the [V] basis and shifts Δ^*L*^_[L]_ **r**_*n*_ owing to libration in the [L] basis. Here, we are working in a linear approximation such that rotation angles are on the order of 0.1 rad. For each particular set of generated shifts, they are transformed into the [M] basis as Δ^*V*^_[M]_ **r**_*n*_ and Δ^*L*^_[M]_ **r**_*n*_ and their sum,
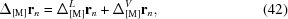
is applied to the corresponding atoms. Details of model generation are discussed in the next sections. This procedure is repeated independently multiple times, leading to structural models distributed according to the TLS matrices.

### 7.2. Calculation of the model shift owing to libration   

Let us suppose that we know (in the basis [M]) the direction of the three mutually orthogonal axes **l**_*x*_, **l**_*y*_, **l**_*z*_ for independent libration as well as the coordinates of the points **w** ^*lx*^_[M]_, **w** ^*ly*^_[M]_, **w** ^*lz*^_[M]_ belonging to each axis. We recalculate the coordinates of these points and the coordinates (*x*_[M]*n*_, *y*_[M]*n*_, *z*_[M]*n*_), *n* = 1, 2, …, *N*, of all atoms **r**_[M]*n*_ of the group into the [L] basis as
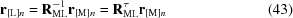
(similar relations are derived for the points **w** ^*lx*^_[M]_, **w** ^*ly*^_[M]_, **w** ^*lz*^_[M]_). We remind the reader that the squared libration amplitudes 〈*d_x_* ^2^〉 = *L*_[L]*xx*_ = λ_1_, 〈*d_y_* ^2^〉 = *L*_[L]*yy*_ = λ_2_, 〈*d_z_* ^2^〉 = *L*_[L]*zz*_ = λ_3_ (§[Sec sec3.2]3.2) and the screw parameters *s_x_*, *s_y_*, *s_z_* (§[Sec sec4.5]4.5) are independent of the basis.

For an atom at a distance *R* = 1 Å from the rotation axis, the probability of the shifts *d_x_*, *d_y_*, *d_z_*, which are numerically equal to the rotation angle in radians, are equal to
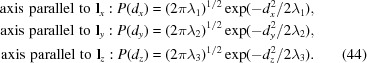
If one of the eigenvalues is equal to 0 then the corresponding *d* is equal to 0 with unit probability. The particular values of *d*_*x*0_, *d*_*y*0_, *d*_*z*0_ are obtained using a random-number generator with an underlying normal distribution (44).

For each of the axes **l**_*x*_, **l**_*y*_, **l**_*z*_ for each atom *n* described by the vector **r**_*n*_, we calculate the coordinates, in the [L] basis, of its shifts Δ^*lx*^_[L]_ **r**_*n*_, Δ^*ly*^_[L]_ **r**_*n*_, Δ^*lz*^_[L]_ **r**_*n*_ owing to the corresponding rotations by *d*_*x*0_, *d*_*y*0_, *d*_*z*0_ (Appendix *A* [App appa]). The overall shift owing to libration around the three axes is the sum
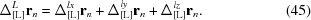
It changes from one atom of the group to another and must be calculated for all atoms of the group with the same (*d*_*x*0_, *d*_*y*0_, *d*_*z*0_) values for a particular instance of the three rotations.

To transform the atomic shift (45) from the [L] basis into the initial [M] basis, we invert (43),
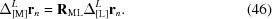



### 7.3. Calculation of the model shift owing to vibration   

In the harmonic approximation, the independent vibration shifts *t_x_*, *t_y_*, *t_z_* expressed in the [V] basis are distributed accordingly to the probability laws
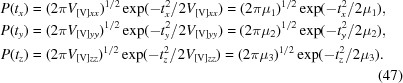
Using a random-number generator, for each model we obtain particular values of *t*_*x*0_, *t*_*y*0_, *t*_*z*0_ using (47). If one of the eigenvalues μ is equal to zero, the zero value is assigned to the corresponding shift. The overall translational shift, common to all atoms of the rigid group, is equal to
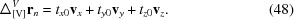
In order to obtain this shift in the [M] basis, we calculate, similarly to (46),
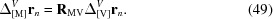



### 7.4. Validation and application to GpdQ   

**Figure 4:**
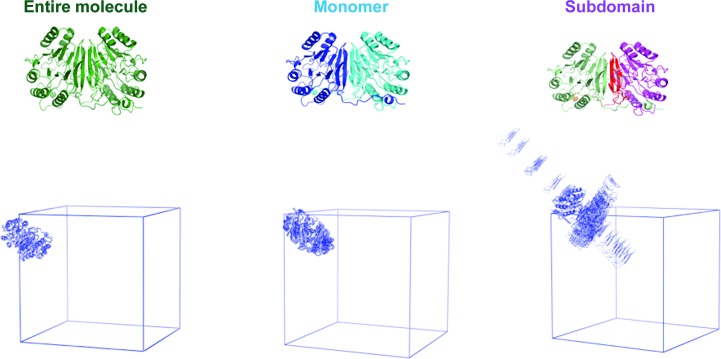
GpdQ TLS ensembles. The GpdQ TLS groups are projected onto the protein structure. The corresponding ensembles produced by *phenix.tls_as_xyz* are shown below. Each TLS PDB ensemble is shown as a single asymmetric unit outlined by the unit cell. An increase in overall motion is apparent going from left to right. The 20-member ensemble is shown for visual simplicity.

**Figure 5:**
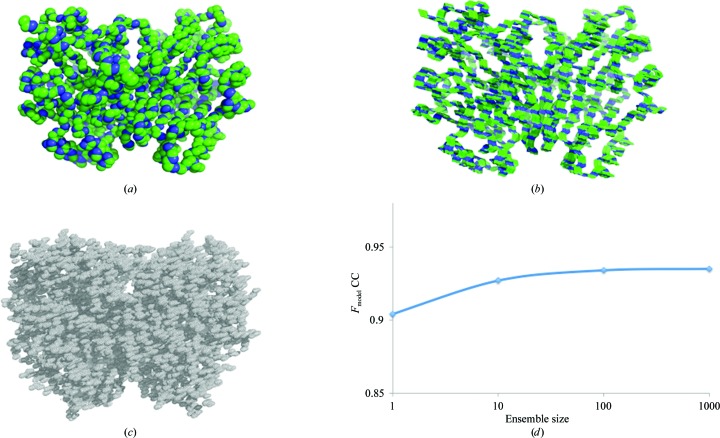
*phenix.tls_as_xyz* ensembles replicate TLS anisotropic motion. (*a*) GpdQ backbone with thermal ellipsoid representation of ‘entire molecule’ TLS anisotropic *B* factors. (*b*) *phenix.tls_as_xyz* ensemble backbones produced from ‘entire molecule’ TLS refinement. (*c*) Complete electron density predicted by ‘entire molecule’ TLS refinement. (*d*) Global correlation coefficient between experimental structure-factor amplitudes *F*_obs_ of the original GpdQ ‘entire motion’ refinement and *phenix.tls_as_xyz* ensembles of various sizes. Convergence values plateau at 0.935.

We generated the ensembles produced by alternative TLS refinements of the glycerophosphodiesterase GpdQ (Jackson *et al.*, 2007 ▸). GpdQ is found in *Enterobacter aerogenes* and contributes to the homeostasis of the cell membrane by hydrolyzing the 3′–5′ phosphodiester bond in glycerophos­phodiesters. Each dimer contains three distinct domains per monomer: an α/β sandwich fold containing the active site, a domain-swapped active-site cap and a novel dimerization domain comprised of dual-stranded antiparallel β-sheets connected by a small β-sheet. Owing to the high global *B* factors and the presence of diffuse signal (Fig. 4 ▸), Jackson *et al.* (2007 ▸) performed three separate TLS refinements to model the crystalline disorder: entire molecule, monomer and subdomain. All TLS refinement attempts improved the *R*_free_ values when compared with the standard isotropic *B*-factor refinement; however, there was no significant difference among the final *R*_free_ values from the various TLS runs. We hypothesized that the diffuse scattering produced by each TLS motion would contain significant differences, as diffuse signal is a direct result of correlated motion. The notion that TLS refinement produces unique diffuse signal has been suggested previously (Tickle & Moss, 1999 ▸). Physical ensembles of the TLS motion, rather than a mathematical description, were required to generate three-dimensional diffuse scattering maps from *phenix.diffuse*. Visual inspection confirmed that the ensembles produced by *phenix.tls_as_xyz* replicated the anisotropic motion predicted by TLS thermal ellipsoids (Fig. 5 ▸). Additionally, we calculated the structure factors predicted by the original TLS refinement ‘entire molecule’ and compared them with the *F*_model_ values (for example, as defined in Afonine *et al.*, 2012 ▸) produced by various *phenix.tls_as_xyz* ensemble sizes. The structure factors converged to a global correlation value of 0.965, demonstrating that *phenix.tls_as_xyz* ensembles accurately represent the motions predicted by TLS refinement. Physical representation of the underlying motion also revealed that while two of the TLS refinements produced motion with small variances (a necessity within TLS theory), using each functional region as a TLS group produced fluctuations that are clearly improbable (Fig. 4 ▸). Thus, viewing TLS refinement in the form of a structural ensemble is a valuable check of the validity of the results, as matrix elements that satisfy the previously described conditions may still produce motions that are clearly implausible.

## 8. Discussion   

While our previous review on the subject (Urzhumtsev *et al.*, 2013 ▸) described the computational details of obtaining the TLS matrices from a known set of vibration and libration parameters (including the position of the axes and correlation of these motions), the current work focuses on the opposite problem of extracting these parameters from a given set of TLS matrices. The problem is not as simple as it may at first seem.

This difficulty arises because current structure-refinement programs vary the matrix elements as independent parameters and often ignore critical constraints on real-space motions. A second challenge is that identical motions may be represented by different vibration–libration combinations. As a consequence, there is no one-to-one relationship between these parameters and the set of TLS matrices. In particular, the traditional way of choosing the matrix **S** so that its trace is equal to zero may result in a mutually inconsistent combination of TLS matrices.

This manuscript describes the constraints that can be used to validate a given set of **T**, **L** and **S** matrices and to improve the refinement of TLS parameters. Beyond the well known conditions of non-negativity for the eigenvalues of **T** and **L**, we also discuss the conditions that relate the matrices, a crucial step in ensuring that the results of TLS refinement correspond to physically possible combinations of librations and vibrations. Taking all these conditions into account provides the possibility of correcting TLS matrices in some cases, if needed. Building these conditions into refinement protocols can eliminate nonplausible refined TLS matrices

The TLS matrix representation is a convenient way of encoding concerted motions into a form suitable for the calculation of structure factors and, in turn, structure refinement. There are two drawbacks to the standard implementation of this method. Firstly, TLS matrices cannot readily be interpreted in terms of underlying motions, but rather require additional processing in order for this information to be extracted. Secondly, direct refinement of the TLS matrix elements may result in refined matrices that cannot be represented as a structural ensemble. To address these two drawbacks, we propose using the set of vibration and libration parameters as refinable variables (an ongoing project for the authors) and reporting them in the PDB files. Indeed, using actual motion descriptors as refinement variables will allow more effective application of physical constraints and in turn guarantee that refined values can be translated to structural ensembles, simplifying the analysis of refinement results, as they will be readily available for interpretation. Finally, this strategy will reduce the chance of overfitting data with atomic models that represent implausible concerted motions.

The survey of PDB entries refined with TLS revealed that roughly 85% of these deposited models contain matrices that are not consistent with the underlying physical model of the concerted motions. Therefore, these matrices cannot be interpreted in terms of rigid-body rotations and translations, and in turn cannot represent these motions (Table 1 ▸). This highlights two urgent needs. Firstly, existing refinement programs should be updated so that they apply appropriate restraints or constraints on refinable parameters of the TLS model. This should be followed by the implementation and use of comprehensive validation of TLS refinement results.

The utility of our presented algorithm is twofold: it validates TLS matrices to confirm that they can represent concerted structural motions and interprets TLS matrices in terms of the elemental motions that they describe. The information about atomic group motions conveyed by the TLS model can be used to analyze possible molecular mechanisms (as illustrated previously). Descriptions of TLS motion may also be used to generate an ensemble of molecular conformations, from which the predicted diffuse scattering signal can be calculated (see the accompanying paper by Van Benschoten *et al.*, 2015 ▸.).

The current procedures for analyzing and validating TLS parameters, as well as the algorithm for generating a set of models from given libration and vibration parameters, are implemented in the *PHENIX* suite and are called *phenix.tls_analysis* and *phenix.tls_as_xyz*, respectively. The programs are available starting with version dev-1890.

## Figures and Tables

**Table 1:** Number of PDB entries in which at least one of the physical conditions on TLS matrices is broken The statistics are shown for the matrices in the PDB (25904 entries with TLS matrices from a total number of 106761 entries as of March 2015) with the default condition tr(**S**) = 0 (upper line) and with the optimal choice of the diagonal **S** elements whenever possible as described in [Sec sec3]3 and [Sec sec4]4 (bottom line). The conditions are, from left to right: matrices **T** and **L** are positive semidefinite (**T** 0 and **L** 0); an absence of libration around one of the axes requires the corresponding elements of the **S** matrix to be equal to 0 (*s* = 0 and *w* = 0); matrix **T** is positive semidefinite after the contribution owing to the displacement of libration axes is removed (**T**_C_ 0); elements of the **S** matrix are limited by the corresponding elements of the **T** and **L** matrices according to the Cauchy conditions (**S** **TL**); the residual *V* matrix is positive semidefinite (**V** 0). The column (**V** 0) includes all conditions from [Sec sec4.3]4.3 and [Sec sec4.4]4.4. When one of the conditions was broken further conditions were not checked.

			Conditions broken							
Mode	Total No. of PDB entries	Total No. of TLS	**T** 0 and **L** 0	*s* = 0 and *w* = 0	**T**_C_ 0	**S** **TL**	**V** 0	Total No. of TLS broken	Total No. of TLS OK	Total No. of PDB entries broken
*t*_S_ = 0	25904	203261	71362	3104	52254	n/a	10492	137212	66049	22707
Best *t*_S_	25904	203261	71362	3104	52255	133	3776	130630	72631	22201

**Table 2:** Examples of the TLS matrices The matrix elements extracted from the PDB files after refinement ([Sec sec6]6).

PDB code	Chain, residue No.	**T** (^2^)	**L** (deg^2^)	**S** (deg)
1dqv	*A*1*A*97	0.1777 0.0090 0.0044	1.4462 0.0160 0.2656	0.0467 0.0523 0.0566
		0.0090 0.1306 0.0019	0.0160 1.2556 0.4713	0.1010 0.0032 0.0164
		0.0044 0.0019 0.1372	0.2656 0.4713 0.8689	0.0090 0.0188 0.0560
	*B*1*B*97	0.1777 0.0090 0.0044	1.4462 0.0160 0.2656	0.0467 0.0523 0.0566
		0.0090 0.1306 0.0019	0.0160 1.2556 0.4713	0.1010 0.0032 0.0164
		0.0044 0.0019 0.1372	0.2656 0.4713 0.8689	0.0090 0.0188 0.0560
1exr	*A*2*A*30	0.0899 0.0040 0.0004	1.3491 0.3760 0.3971	0.0249 0.3537 0.0874
		0.0040 0.1333 0.0058	0.3760 0.6103 0.3389	0.1275 0.0783 0.0144
		0.0004 0.0058 0.0728	0.3971 0.3389 0.3698	0.0183 0.0542 0.0103
	*A*31*A*74	0.0925 0.0037 0.0041	0.3464 0.3638 0.2923	0.0220 0.0419 0.0793
		0.0037 0.0673 0.0062	0.3638 0.3283 0.1212	0.0061 0.0018 0.1161
		0.0041 0.0062 0.1119	0.2923 0.1212 0.3799	0.0041 0.0385 0.0009
	*A*75*A*84	0.2433 0.0144 0.0917	0.0736 0.0171 0.0565	0.4357 0.1151 0.2346
		0.0144 0.2867 0.1720	0.0171 0.0068 0.0203	0.2521 0.3549 0.2041
		0.0917 0.1720 0.1749	0.0565 0.0203 0.0336	0.3793 0.1499 0.0111
	*A*85*A*147	0.0747 0.0110 0.0066	0.6097 0.0786 0.1864	0.0180 0.1466 0.0378
		0.0110 0.1384 0.0062	0.0786 0.6474 0.6233	0.0155 0.0872 0.0542
		0.0066 0.0062 0.0673	0.1864 0.6233 0.9637	0.0440 0.1022 0.0852
4b3x	*A*1*A*65	0.4663 0.0991 0.0764	0.4738 0.0063 0.2318	0.0391 0.0307 0.4316
		0.0991 0.5443 0.0321	0.0063 0.2120 0.0584	0.0587 0.1786 0.2003
		0.0764 0.0321 0.5001	0.2318 0.0584 0.1312	0.3665 0.4293 0.0403
	*A*66*A*363	0.1649 0.0259 0.0184	0.8808 0.0912 0.1736	0.0345 0.0102 0.0661
		0.0259 0.1422 0.0055	0.0912 0.9522 0.0972	0.1159 0.0222 0.0999
		0.0184 0.0055 0.2028	0.1736 0.0972 1.6563	0.0424 0.1330 0.0237

**Table 3:** Examples of parameters of the elemental motions found from decomposition of the TLS matrices The parameters are given in the units used in this article, allowing an easy estimation of the corresponding atomic displacements. The directions of the libration and rotation axes are not given.

PDB code	Chain, residue No.	**T**: *t*_*x*_, *t*_*y*_, *t*_*z*_ ()	**L**: *d*_*x*_, *d*_*y*_, *d*_*z*_ (rad)	**S**: *s*_*x*_, *s*_*y*_, *s*_*z*_ ()	tr(**S**)
1dqv	*A*1*A*97	0.3455 0.3671 0.4172	0.01239 0.02044 0.02273	1.343 1.137 1.319	0
	*B*1*B*97	0.3634 0.3885 0.4166	0.01608 0.01753 0.03069	0.679 1.177 0.200	0
1exr	*A*2*A*30	0.1944 0.2663 0.2870	0.00000 0.01602 0.02182	0.000 2.951 3.408	>0
	*A*31*A*74	0.2110 0.2939 0.3068	0.00000 0.00860 0.01637	0.000 18.14 5.028	0
	*A*75*A*84	0.1692 0.4906 0.6598	0.00000 0.00000 0.00000	0.000 0.000 0.000	0
	*A*85*A*147	0.0002 0.2270 0 3078	0.00553 0.01418 0.02109	20.83 0.800 1.672	0
4b3x	*A*1*A*65	0.0994 0.6064 0.7116	0.00000 0.00825 0.01343	0.000 2.718 11.05	0
	*A*66*A*363	0.3306 0.4102 0.4413	0.01568 0.01720 0.02283	3.164 2.276 0.197	0

## Appendix A Technical details of the algorithm

### A1. Definition of the transition matrices

Let us have three mutually orthogonal unit vectors **l**_*x*_, **l**_*y*_, **l**_*z*_ described respectively by their coordinates [(*l_x_*)_[M]*x*_, (*l_x_*)_[M]*y*_, (*l_x_*)_[M]*z*_], [(*l_y_*)_[M]*x*_, (*l_y_*)_[M]*y*_, (*l_y_*)_[M]*z*_], [(*l_z_*)_[M]*x*_, (*l_z_*)_[M]*y*_, (*l_z_*)_[M]*z*_] in the Cartesian basis [M]. These vectors can be considered as a new basis [L]. The coordinates of a vector **r** in [L] and [M] are expressed through each other using the transition matrix **R**_ML_ as

Transition matrices for other pairs of bases, for example from [V] to [L] (§[Sec sec2.1]2.1), [M] to [V] and *vice versa* (§[Sec sec7.3]7.3) are defined in a similar way.

### A2. Cauchy conditions on the elements of the TLS matrices

Let *d_x_*, *d_y_*, *d_z_* and *u_x_*, *u_y_*, *u_z_* be random displacements owing to rotations and translations, respectively. Since *S_xx_* = 〈*d_x_* *u_x_*〉, *V_xx_* = 〈*u_x_* *u_x_*〉, *L_xx_* = 〈*d_x_* *d_x_*〉 (Schomaker & Trueblood, 1968 ▸; see also equations 8.5–8.7 in Urzhumtsev *et al.*, 2013 ▸), it follows from the Cauchy inequality that

In the basis [L] with **S**_[L]_ = **S**_C_(*t*_S_) (18), condition (51) becomes

Similarly, we obtain two other conditions

### A3. Polynomials for the coefficients of the characteristic equation

If *t_xx_*, *t_xy_*, *t_xz_* *etc.* are respective elements of the matrix **T**_Λ_ (29), the coefficients (36) of the characteristic equation as functions of the parameter *t* are

### A4. Explicit expression for the atomic shifts owing to rotations with given parameters

Let (*x*_[L]_, *y*_[L]_, *z*_[L]_) be the Cartesian coordinates of a point **r** in the basis [L]. For a rotation around the axis parallel to **l**_z_ and crossing the point **w** ^*lz*^_[L]_ = (*w* ^*lz*^_[L]*x*_, *w* ^*lz*^_[L]*y*_, *w* ^*lz*^_[L]*z*_), we first recalculate the coordinates of the vector **r** − **w** ^*lz*^_[L]_ with respect to the rotation axis,

If **r**′ stands for the position of the same point after rotation by angle *d*_*z*0_ around this axis, the coordinates of **r**′ − **w** ^*lz*^_[L]_, the point with respect to the axis, are

This gives the atomic shift

There are similar expressions for the shift owing to rotations around the other two axes:

## Appendix B List of abnormal situations requiring interruption of the procedure

This appendix summarizes the situations when the described algorithm breaks. Each condition below starts from the corresponding program message and then refers to the main text and to Fig. 1 ▸. To analyze the PDB content, the program can be run in a special regime when at step *C* we assign *t*_S_ = 0, *i.e.* when the matrix **S** is taken without any correction [in most cases this corresponds to the current default constraint tr(**S**) = 0]. In this regime, we directly calculate the matrices **C** and check the conditions (x)–(xii).

*Step *A*: basis [L]; determination of the libration axes and amplitudes.*

(i) ‘Input matrix L[M] is not positive semidefinite’, §[Sec sec3.1]3.1.

(ii) ‘Input matrix T[M] is not positive semidefinite’, §[Sec sec3.1]3.1.

*Step *B*: determination of the points **w** at the libration axes.*

(iii) ‘Non-zero off-diagonal S[L] and zero L[L] elements’, §[Sec sec3.2]3.2, (15).

(iv) ‘Matrix T_C[L] is not positive semidefinite’, §[Sec sec3.2]3.2, (17).

*Step *C*: determination of the screw parameters: left branch (librations around all three axes).*

(v) ‘Empty (tmin_c, tmax_c) interval’, §[Sec sec4.2]4.2, (23). *t*_min,C_ > *t*_max,C_.

(vi) ‘Empty (tmin_t, tmax_t) interval’, §[Sec sec4.3]4.3, (31). *t*_min,τ_ > *t*_max,τ_.

(vii) ‘Negative argument when estimating tmin_a’, §[Sec sec4.3]4.3, (38).

(viii) ‘Intersection of the intervals for t_S is empty’, §[Sec sec4.3]4.3, step (ii). *t*_min_ > *t*_max_.

(ix) ‘t_min = t_max giving non positive semidefinite V_lambda’, §[Sec sec4.3]4.3, step (iii).

(x) ‘Interval (t_min, t_max) has no t value giving positive semidefinite V’, §[Sec sec4.3]4.3, step (iv).

*Step *C*: determination of the screw parameters: right branch (no libration around at least one of the axes).*

(xi) ‘Cauchy-Schwarz conditions are wrong for the found t_S’, (22) with *t*_S_ calculated in §[Sec sec4.4]4.4.

(xii) ‘Non-zero diagonal S[L] element for a zero L[L] element’, §[Sec sec4.4]4.4.

*Step *D*: determination of the vibration parameters.*

(xiv) ‘Matrix V[L] is not positive semidefinite’, §[Sec sec5.1]5.1.

*Extra checks at step *C* when some conditions may fail owing to rounding errors.*

(1) When calculating square roots in (24), the arguments are non-negative by previous conditions (i) and (iv) since the diagonal elements of a positive semidefinite matrix are non-negative.

(2) When calculating square roots in (28), the arguments are non-negative by previous condition (i).

(3) When calculating square roots in (31), the argument τ_max_ is non-negative since the eigenvalues of **T**_C[L]_ are also non-negative by previous condition (iv).
