# From deep TLS validation to ensembles of atomic models built from elemental motions. Addenda and corrigendum

**DOI:** 10.1107/S2059798316013048

**Published:** 2016-08-31

**Authors:** Alexandre Urzhumtsev, Pavel V. Afonine, Andrew H. Van Benschoten, James S. Fraser, Paul D. Adams

**Affiliations:** a Centre for Integrative Biology, Institut de Génétique et de Biologie Moléculaire et Cellulaire, 1 rue Laurent Fries, BP 10142, 67404 Illkirch, France; b Faculté des Sciences et Technologies, Université de Lorraine, BP 239, 54506 Vandoeuvre-les-Nancy, France; c Molecular Biophysics and Integrated Bioimaging Division, Lawrence Berkeley National Laboratory, Berkeley, California, USA; d Department of Bioengineering and Therapeutic Sciences, University of California, San Francisco, San Francisco, California, USA; e Department of Bioengineering, University of California, Berkeley, Berkeley, California, USA

**Keywords:** TLS model, TLS matrices, ensemble of atomic models, atomic displacement matrices, model validation, addenda and corrigendum

## Abstract

Researcher feedback has indicated that in Urzhumtsev *et al.* [(2015) *Acta Cryst.* D**71**, 1668–1683] clarification of key parts of the algorithm for interpretation of TLS matrices in terms of elemental atomic motions and corresponding ensembles of atomic models is required. Also, it has been brought to the attention of the authors that the incorrect PDB code was reported for one of test models. These issues are addressed in this article.

## Abstract 2

Some key parts of the algorithm for interpretation of TLS matrices in terms of elemental atomic motions and corresponding ensembles of atomic models described in the article by Urzhumtsev *et al.* [(2015) *Acta Cryst.* D**71**, 1668–1683] are clarified and developed, and a reference on a wrong model is corrected.

## 1. Incorrect PDB code   

In the original article (Urzhumtsev *et al.*, 2015 ▸), we used several atomic models in order to test the algorithms and provide examples. Unfortunately, the incorrect PDB code had been reported for one of them. Everywhere in the text (§6.2, Tables 2 and 3), 1rge should be used instead of 1dqv and ribonuclease S should be used instead of synaptotagmin. We apologize for this confusion. The diffraction data set used for test refinement of ribonuclease S was obtained from the *CCP*4 (Winn *et al.*, 2011 ▸) distribution (http://www.ccp4.ac.uk/examples/rnase/rnase25.mtz).

## 2. Origin choice   

The problem of the origin choice is discussed in detail in the review of Urzhumtsev *et al.* (2013 ▸) leading us to provide less detail in Urzhumtsev *et al.* (2015 ▸). As mentioned in §2.2 of Urzhumtsev *et al.* (2015 ▸), the **T** and **S** matrices depend on the point (origin of the TLS group) with respect to which the three libration axes are defined. This is also important for generating the 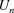 matrices from a set of TLS matrices. Confusion arises from the fact that the TLS origin may be, and in fact usually is, different from the origin of the coordinate system in which the atomic coordinates are provided.

The choice of the TLS origin is arbitrary; some typical choices are described in §2.2. Let 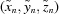, *n* = 1,…*N* be atomic Cartesian coordinates as the input parameters of the procedure; for example, they may be the coordinates given in the PDB file. Let 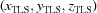 be respective coordinates of the origin of the TLS group. The origin of basis [M] defined in Urzhumtsev *et al.* (2015 ▸) is assumed to coincide with this point. This means that the coordinates that are input to the ensemble generating procedure (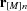 in §7.2 of Urzhumtsev *et al.*, 2015 ▸) are also expected to be shifted to the origin of the TLS group as follows 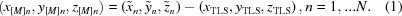
The matrix
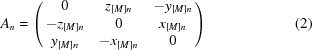
[equation (3) in Urzhumtsev *et al.*, 2015 ▸] uses these new coordinates (1).

## 3. *U*_*n*_ matrices   

The TLS model is valid for harmonic motions and, as a consequence, for small libration amplitudes only. It allows for calculation of the individual atomic displacement parameters 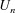 in two different ways. They may be calculated analytically using the formulae (2) and (3) from Urzhumtsev *et al.* (2015 ▸). Alternatively, the same matrices can be calculated numerically from the coordinates of the set of models generated explicitly using the procedure described in §7 and *Appendix A* of Urzhumtsev *et al.* (2015 ▸). We stress that in formulae (59)–(61) the expressions 
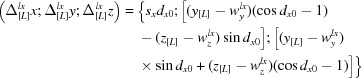


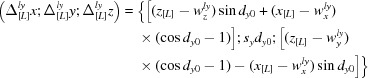


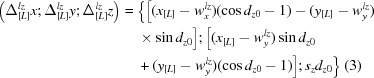
are the coordinates of the libration shifts in the basis [L]; similarly, the values 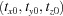 in (48) are the coordinates of the vibration shifts in the basis [V]. These coordinates must be converted into the basis [M] in order to obtain the coordinates of the total shifts 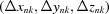, *n* = 1,…*N*, to be applied to the atomic coordinates 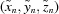, *n* = 1, … *N*. Here *k* is the number of the generated model.

Once an ensemble is generated, the atomic displacement matrix 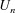 for each atom *n*

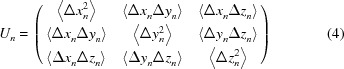
can be calculated directly from the coordinates 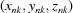, *k* = 1,…*K* of the multiple copies of the same atom in the ensemble [see formula (2.2) from Urzhumtsev *et al.*, 2013 ▸]. Here atomic coordinates are in the basis [M] and averaging is performed over all *K* instances of the atom in the ensemble.

These two somewhat independent routes to obtain the 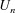 matrices allow a convenient way of validating the described procedures. Indeed, given parameters of elemental motions one can construct TLS matrices and then calculate 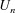 from these TLS matrices using the analytical expression cited above. Also, one can use TLS matrices to generate an ensemble of models and then derive 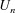 from the ensemble using formula (4). We added this comparison to *cctbx* (Grosse-Kunstleve *et al.*, 2002 ▸) as a test-exercise of the implementation.

Now the *Phenix* (Adams *et al.*, 2010 ▸) command phenix.tls_as_xyz model.pdb n_models = N creates three PDB files containing the following.

(i) An ensemble of *N* models (*N* can be any positive integer) that are consistent with the TLS model (TLS records must be present in the model.pdb file header).
(ii) A single model with anisotropic 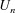 (*U_TLS_* in ANISOU records) calculated from the TLS matrices analytically.
(iii) A single model with anisotropic 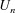 (*U*_ensemble_, ANISOU records) calculated numerically from the ensemble of models.

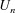
 obtained using the two different approaches are expected to be similar with possible differences arising from several sources, such as the following.

(*a*) Non-linearity of the TLS approximation (large libration amplitudes; Table 1 ▸).
(*b*) A finite number of models in the ensemble; empirically we found that 5000–10 000 is sufficient most of the time (Fig. 1 ▸).
(*c*) Numerical errors arising from a long chain of transformations: from decomposing TLS matrices into basic parameters of elemental motions and using these parameters to generate a large set of models that are then used to compute the anisotropic 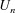 matrices.

Since TLS modeling is based on a linearity approximation (Urzhumtsev *et al.*, 2013 ▸) one may expect a significant difference between the matrices 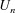 calculated analytically using TLS matrices and those calculated directly by (4) if the libration amplitudes are large. As mentioned in §6.1 of Urzhumtsev *et al.* (2015 ▸), large values for the vibration and libration amplitudes are not physically meaningful.

Table 1 ▸ shows matrices 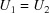 for an artificial example of two atoms with the coordinates (0, 0, 0) and (1, 2, 3) when the only motion applied was libration around the axis parallel to **Oz** that passes through the center of mass of this system taken as the TLS origin. In the basis [M] the coordinates of these atoms are equal to (−0.5, −1.0, −1.5) and (0.5, 1.0, 1.5), respectively. The matrices were obtained using the two methods discussed above applying different amplitudes *dz* (for definitions see Urzhumtsev *et al.*, 2015 ▸). As discussed previously (§2.3 in Urzhumtsev *et al.*, 2013 ▸, and references therein), the discrepancy between two corresponding matrices is significant when the libration amplitude becomes larger than approximately 0.10–0.15 rad (6–9°).

To investigate the number of models sufficient to reproduce *U*_TLS_ by *U*_ensemble_, we took the C_A_ atoms from fragment A6–A61 of protein G IgG-binding domain III model (PDB code 2igd) and fitted TLS matrices to individual anisotropic 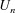 of this model using the *phenix.tls* tool. Then we calculated *U*_TLS_ for each atom of the model and independently generated a set of random models from which we calculated *U*_ensemble_ to compare them with *U*_TLS_. Fig. 1 ▸ shows the mean relative difference between the sets of the *U* elements as a function of the number of generated models.

## Figures and Tables

**Figure 1:**
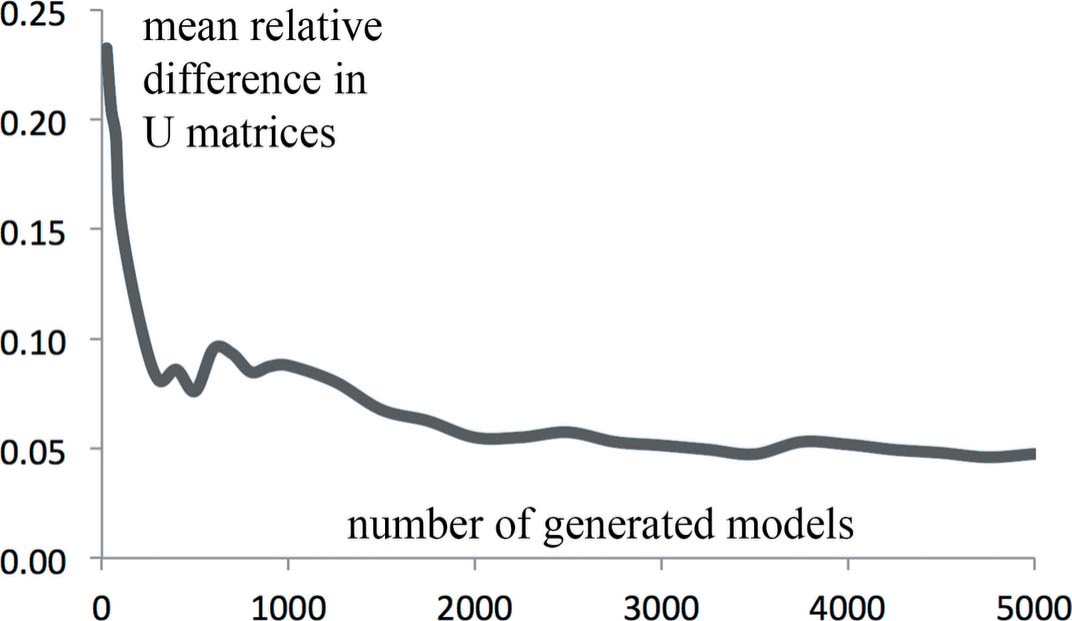
Comparison of the matrices *U*_TLS_ calculated for an atomic model analytically (see the text for details) and the matrices calculated from the ensemble models (*U*_ensemble_) as a function of the number of models generated. The mean relative difference between the elements of the two sets of matrices is defined as 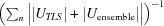 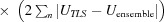 where the sums are calculated over all atoms and over all six elements of each pair of *U* matrices.

**Table 1:** Matrix *U* calculated analytically for two atoms (see the text for details) from the TLS matrices (*U*_TLS_) and numerically from an ensemble of 20 000 models (*U*_ensemble_) for different libration amplitude *dz* Matrix elements *U*_*zz*_, *U*_*xz*_, *U*_*yz*_ are always equal to 0 and not shown. The last column shows the ratio of the maximal difference between respective *U*_TLS_ and *U*_ensemble_ elements compared to the maximal *U*_TLS_ element. Two lines for *dz* = 0.10 stand for two independent runs of the random model generation.

*dz* (rad/°)	*U*_TLS_ (*U*_*xx*_, *U*_*yy*_, *U*_*xy*_)	*U*_ensemble_ (*U*_*xx*_, *U*_*yy*_, *U*_*xy*_)	Δ_max_/*U*_max_
0.05/2.9	0.00250, 0.00063, −0.00125	0.00250, 0.00063, −0.00125	0
0.07/4.0	0.00490, 0.00123, −0.00245	0.00487, 0.00122, −0.00242	0.006
0.09/5.2	0.00810, 0.00202, −0.00405	0.00791, 0.00201, −0.00394	0.023
0.10/5.7	0.01000, 0.00250, −0.00500	0.00993, 0.00254, −0.00496	0.007
0.10/5.7	0.01000, 0.00250, −0.00500	0.00993, 0.00253, −0.00494	0.007
0.15/8.6	0.02250, 0.00562, −0.01125	0.02207, 0.00567, −0.01079	0.020
0.20/11.5	0.04000, 0.01000, −0.02000	0.03802, 0.01025, −0.01861	0.049
0.25/14.3	0.06250, 0.01562, −0.03125	0.05970, 0.01664, −0.02858	0.045
0.30/17.2	0.09000, 0.02250, −0.04500	0.08204, 0.02415, −0.03909	0.088
0.50/28.6	0.25000, 0.06250, −0.12500	0.20432, 0.07408, −0.08651	0.183
0.70/40.1	0.49000, 0.12250, −0.24500	0.32987, 0.15339, −0.11880	0.327
0.90/51.6	0.81000, 0.20250, −0.40500	0.44468, 0.25448, −0.12348	0.451
